# *Impatiens longcanggouensis* (Balsaminaceae): a new species from Sichuan, China

**DOI:** 10.3897/phytokeys.270.174879

**Published:** 2026-01-26

**Authors:** Qiang Luo, Hong Chen, Ying Yuan, Hong Yang, Chan Liu, Xinqiang Song, Shen Ma

**Affiliations:** 1 Xichang University, Xichang, Sichuan 615013, China Xichang University Xichang China; 2 Southwest Forestry University, Kunming 650224, China Southwest Forestry University Kunming China; 3 Daxiangling Nature Reserve Management and Protection Center of Yingjing County, Ya’an, Sichuan 625000, China Daxiangling Nature Reserve Management and Protection Center of Yingjing County Ya’an China

**Keywords:** Balsaminaceae, China, *Impatiens longcanggouensis* sp. nov., Sichuan

## Abstract

*Impatiens longcanggouensis* Q.Luo (Balsaminaceae), a new species from Yingjing County, Sichuan Province, China, is described and illustrated. Its morphology, including pollen and seed characters observed via scanning electron microscopy (SEM), is documented in detail. The new species is morphologically similar to *I. recurvicornis*, but is readily distinguished by the following combination of characters: leaf margin shallowly arcuate-serrate (vs. serrulate); abaxial leaf surface usually purplish-red (vs. pale green); inflorescence commonly two-flowered (vs. one-flowered); bracts lanceolate, 2–2.5 mm long (vs. ovate, 3–4 mm long); flowers 1.2–1.8 cm long (vs. 3–4 cm long); dorsal petal 6–8 mm in diameter, with two erect, stout rostella along the abaxial mid-vein (vs. dorsal petal ca. 13 mm in diameter with a beaked apex); lower sepal narrowly funnel-form with a spreading mouth (vs. navicular with an oblique mouth); and capsule clavate (vs. linear).

## Introduction

The genus *Impatiens* (Balsaminaceae), comprising over 1,000 species worldwide, is primarily distributed across the tropical and subtropical mountains of the Eastern Hemisphere (Grey-Wilson 1980; Yu 2012). Its five major diversity centres encompass tropical Africa, Madagascar, southern India and Sri Lanka, the eastern Himalayas and Southeast Asia (Yuan et al. 2004). The genus exhibits remarkable species diversity and complex morphological variation. Nevertheless, its flowers — key diagnostic features — are extremely delicate. Upon preservation as herbarium specimens, floral parts become difficult to separate and reconstruct, making *Impatiens* one of the most taxonomically challenging plant genera (Chen 2001). Consequently, molecular systematics has been increasingly employed to resolve phylogenetic relationships and aid species identification (Yuan et al. 2004; Qiu et al. 2023). Furthermore, pollen grains and seeds, developing within enclosed floral structures, show greater structural conservation. Hence, their micromorphology and surface ornamentation serve as critical characters for interspecific differentiation (Utami and Shimizu 2005; Janssens et al. 2018; Song et al. 2022).

China hosts abundant *Impatiens* resources. The first systematic treatment of Chinese Balsaminaceae recorded 220 species (Chen 2001). By March 2022, this number had increased to 352 (Yuan et al. 2022a), with numerous new species described subsequently, such as *Impatiens nushanensis* Z.Wang, P.P.Wu & S.X.Yu (Wang et al. 2022a), *I. chenmoui* Zheng W.Wang, X.C.Li & Q.Wang (Wang et al. 2022b), *I. bijieensis* X.X.Bai & L.Y.Ren (Ren et al. 2022), *I. liupanshuiensis* X.X.Bai & T.H.Yuan (Yuan et al. 2022b), *I. longyangensis* Y.Y.Cong, G.W.Hu & S.Peng (Hu et al. 2022), *I. yaojiapingensis* Y.Y.Cong, G.W.Hu & T.Hu (Hu et al. 2022), *I. yunlingensis* S.X.Yu, Chang Y.Xia & J.H.Yu (Yu et al. 2022), *I. spiralis* Y.Y.Cong, G.L.Zhang & Y.M.Zheng (Zheng et al. 2023), *I. cavaleriei* X.X.Bai & R.X.Huang (Huang et al. 2023), *I. mogangensis* Y.M.Shui & W.H.Chen (Lyu et al. 2024), *I. beipanjiangensis* Jian Xu & H.F.Hu (Hu et al. 2024), *I. lhunzeensis* J.Tian, G.W.Hu & Q.F.Wang (Tian et al. 2024), *I. uncata* Y.Y.Cong & J.J.Zhou (Qiu et al. 2024), *I. maolanensis* Z.B.Xiong & Q.Y.Wen (Li et al. 2025), *I. fujianensis* Liang Ma, Xin Y.Chen & S.P.Chen (Ma et al. 2024), as well as *I. perforata*, *I. aciformis*, *I. zhui*, *I. kuntsunii* and *I. cordibracteata* (Huang and Liu 2025b).

Southwest China is the distribution centre for *Impatiens* in the country, with Yunnan, Sichuan and Tibet being the three provinces/regions with the highest species diversity, hosting 165, 119 and 69 species, respectively (Luo 2011; Chen et al. 2019; Yuan et al. 2022a). The discovery of new species has continued apace in recent years. For instance, those newly reported from Sichuan include *I. sikaiensis* Q.Luo & Ying Yuan (Yuan et al. 2022c), *I. zhaojueensis* Q.Luo (Luo et al. 2023), *I. yingjingensis* X.Q. Song, B.N. Song & Biao Yang (Song et al. 2024), *I. amphitricha* H.W. Huang & R. Li (Huang et al. 2025a) and *I. meishanensis* K. Huang & Z.X. Fu (Zhang et al. 2025). Notably, endemism is highly prominent in this genus, with an endemic rate of 87.03% for China as a whole and 37.96% specifically for Sichuan Province (Cen et al. 2025).

Longcanggou National Forest Park, located in Yingjing County, Sichuan Province, is a well-preserved ecosystem with minimal human disturbance and high biodiversity, hosting several National Key Protected Wild Plants, such as *Davidia involucrata* Baill., *Taxus wallichiana* var. *chinensis* (Pilg.) Florin and *Cercidiphyllum japonicum* Siebold & Zucc. During a field investigation in August 2025, we identified several *Impatiens* species in the area (including *I. omeiana* Hook.f., *I. wilsonii* Hook.f., *I. microstachys* Hook.f., *I. platychlaena* Hook.f., *I. oxyanthera* Hook.f., *I. tienchuanensis* Y.L.Chen and *I. rostellata* Franch.) alongside an unidentified taxon. Morphological traits of the unknown taxon were documented from fresh material and voucher specimens were prepared. Its pollen and seed micromorphology were examined using scanning electron microscopy (SEM). Through comparison with relevant literature (Chen 2001; Chen et al. 2007; Yu 2012; Suksathan and Ruchisansakun 2022; Cong et al. 2024) and with type specimens of closely-related species (e.g. *I. minimisepala* Hook.f., P00780686; *I. membranifolia* Franch. ex Hook.f., P00780687–P00780690; *I. trigonosepala* Hook.f., P00780744, P00780745), we determined that, although it is morphologically similar to *I. recurvicornis* Maxim. (PE 00078361, PE 00078362), the consistent diagnostic differences warrant its recognition as a new species.

## Materials and methods

The habitat of the new species was examined and its plants, flowers and fruits were photographed. Detailed morphological descriptions were prepared from fresh specimens. Mature seeds and pollen grains were collected from the type specimen, air-dried and mounted on stubs using double-sided adhesive tape. The samples were sputter-coated with gold using a 208HR Sputter Coater (Cressington) and imaged with a Thermo Scientific Apreo 2C SEM. The sizes of 20 randomly selected pollen grains and seeds were measured under an optical microscope (Luo et al. 2014).

## Taxonomic treatment

### Impatiens longcanggouensis

Taxon classificationPlantaeEricalesBalsaminaceae

Q.Luo sp. nov.

A1C87FDB-A8DA-58F3-9BFD-D533E17A84DB

urn:lsid:ipni.org:names:77375586-1

Figs 1, 2, 3

#### Diagnosis.

*Impatiens longcanggouensis* is morphologically similar to *I. recurvicornis*, but is readily distinguished by the following combination of characters: leaf margin shallowly arcuate-serrate (vs. serrulate); abaxial leaf surface usually purplish-red (vs. pale green); inflorescence commonly two-flowered (vs. one-flowered); bracts lanceolate, 2–2.5 mm long (vs. ovate, 3–4 mm long); flowers 1.2–1.8 cm long (vs. 3–4 cm long); dorsal petal 6–8 mm in diameter, with two erect, stout rostella along the abaxial mid-vein (vs. dorsal petal ca. 13 mm in diameter with a beaked apex); lower sepal narrowly funnel-form with a spreading mouth (vs. navicular with an oblique mouth); and capsule clavate (vs. linear).

**Figure 1.**
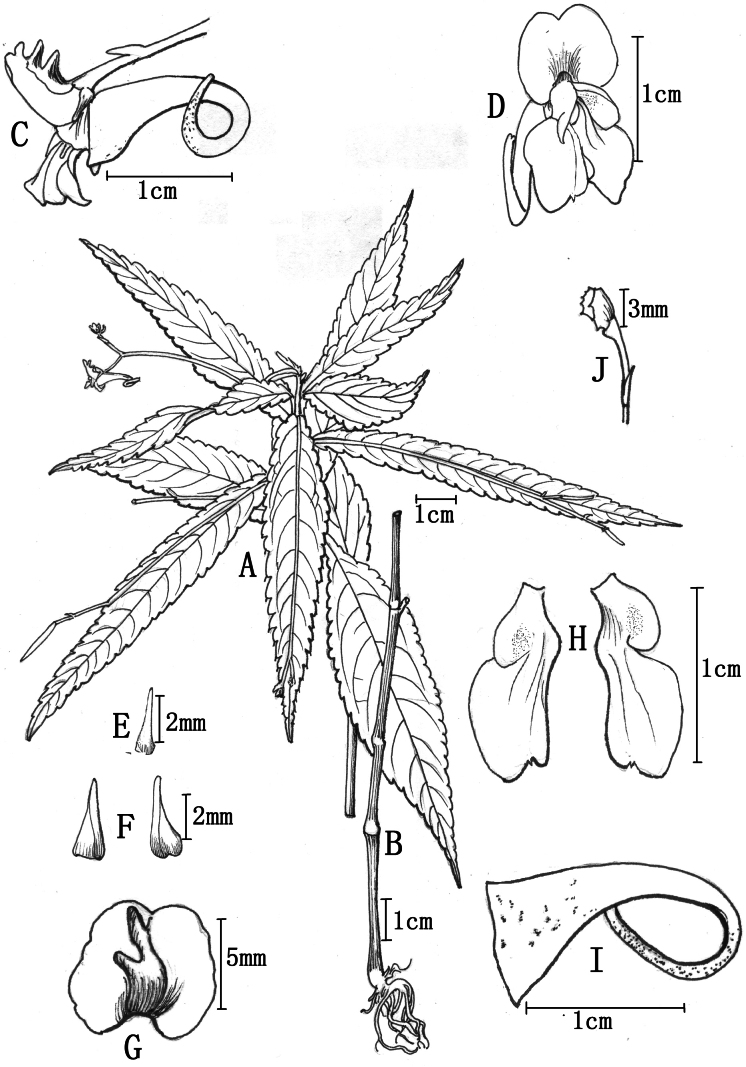
*Impatiens longcanggouensis* sp. nov. **A**. Upper plant; **B**. Lower plant; **C**. Flower, lateral view; **D**. Flower, frontal view; **E**. Bract; **F**. Lateral sepals; **G**. Dorsal petal; **H**. Lateral united petals; **I**. Lower sepal; **J**. Anther. Drawn by Wang Ling from Q. Luo (holotype: PE).

#### Type.

China • Sichuan Province, Yinjing County, Longcanggou National Forest Park, 1,540 m above sea level (a.s.l.), 29°36'41.95088"N, 102°53'48.53844"E, 8 August 2025, *Luo Qiang 25080806* (holotype: PE; isotypes: XIAS). • Same locality, 1,410 m a.s.l., 29°37'38.14587"N, 102°53'15.16814"E, 28 August 2025, Luo Qiang 25082801 (paratype: XIAS).

**Figure 2.**
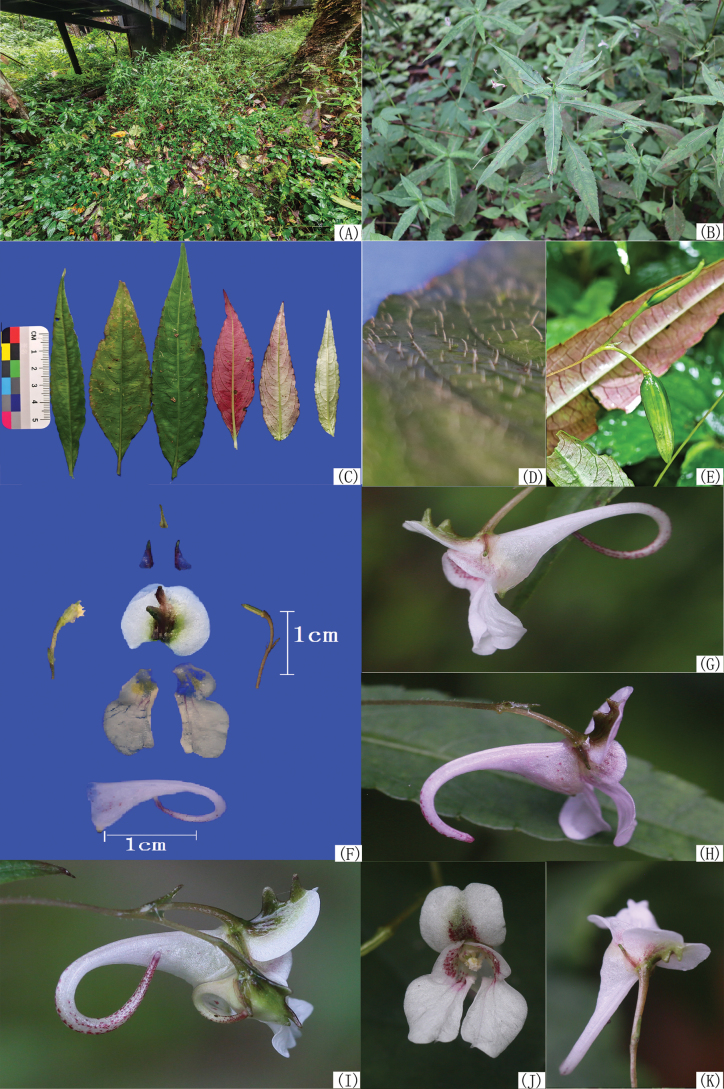
*Impatiens longcanggouensis* sp. nov. **A**. Habitat; **B**. Plant; **C**. Leaves; **D**. Adaxial scabrous hairs; **E**. Capsules; **F**. Flower anatomy; **G–K**. Flowers from different angles.

#### Morphology.

Annual herb, 30–60 cm tall, subglabrous. Stems erect, lower portion exposed, upper portion branched. Leaves alternate; lower leaves with petioles up to 3 cm; blades membranous, lanceolate or ovate-lanceolate, 4–16 cm long, 1–3.5(–4) cm wide; apex long-acuminate or caudate; base cuneate in lower leaves, subtruncate in upper leaves; shallowly arcuate-serrate with mucronate teeth; lateral veins 6–9 pairs; adaxial surface green or dark green, hispid; abaxial surface usually purplish-red, rarely greyish-green. Inflorescences in upper leaf axils, 4–10 cm long, filiform, spreading or curved, often puberulent, two-flowered. Pedicels 4–13 mm long, with a single bract at the base; bracts lanceolate, 2–2.5 mm long, apex beaked, persistent. Flowers white or faintly pinkish, 1.2–1.8 cm long. Lateral sepals 2, green or purplish-red and spotted with purplish-red, ovate or triangular-ovate, 2.5–3.5 mm long, apex beaked or acute, mid-vein sometimes marginal. Dorsal petal suborbicular, 6–8 mm in diameter, emarginate at both ends, with a thickened dorsal mid-rib bearing two erect, stout rostella. Lateral united petals 1–1.5 cm long, subclawed, two-lobed; basal lobes ovate or broadly ovate, ca. 2.5 mm long; distal lobes rhomboid or oblong-dolabriform, 0.8–1.2 cm long, 4–6 mm wide, apically truncated or retuse with a small mucro, dorsal auricle reflexed. Lower sepal 1.2–1.5 cm long, narrowly funnel-shaped, mouth spreading, apex obtuse-acuminate, base gradually narrowed into an incurved long spur. Filaments linear; anthers acute. Ovary linear, erect, five-angled, apex acuminate. Capsule clavate, 1.5–2.3 cm long, seeds 2 to 3.

#### Palynology.

**Figure 3.**
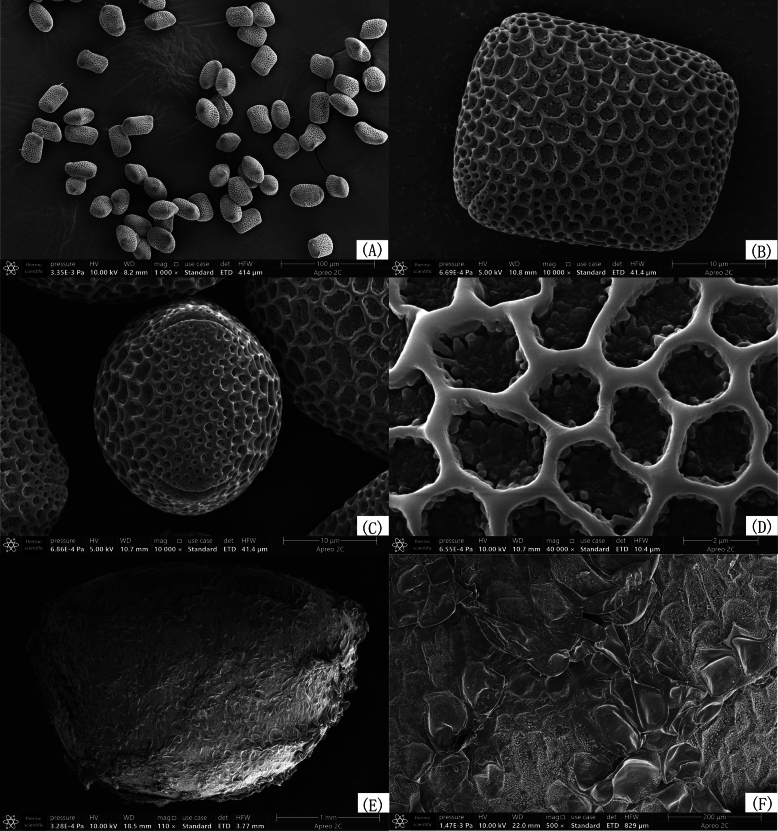
Scanning electron microscopy (EM) micrographs of pollen grains and seed of *Impatiens longcanggouensis* sp. nov. **A–D**. Pollen grains; **E, F**. Seed.

(Fig. 3A–D) Pollen grains four-colpate, oblate-spheroidal or subprolate in equatorial view, subcircular in polar view. Pollen size (equatorial view): 34.83 (30.96–36.77) × 21.28 (17.44–28.80) μm. Exine reticulate; in polar view, lumina smaller with shallower muri, lacking or bearing sparse granules; in equatorial view, lumina uniformly distributed, larger with deeper muri, sparsely covered with irregular granules.

#### Seed micromorphology.

(Fig. 3E, F) Seeds ovoid or ellipsoid, bilaterally compressed, 3.22 (3.02–3.46) × 2.29 (1.98–2.51) mm. Seed coat surface reticulate, with most lumina flat; some testa cells showing slightly raised anticlinal walls and slightly concave outer periclinal walls. The entire surface is irregularly covered with small granules.

#### Etymology.

The epithet *longcanggouensis* refers to the type locality: Longcanggou Yinjing County, Sichuan Province, China. The new species is named ‘龙苍沟凤仙花’ in Chinese.

#### Phenology.

Flowering from August to September; fruiting from August to October.

#### Habitat and distribution.

**Figure 4.**
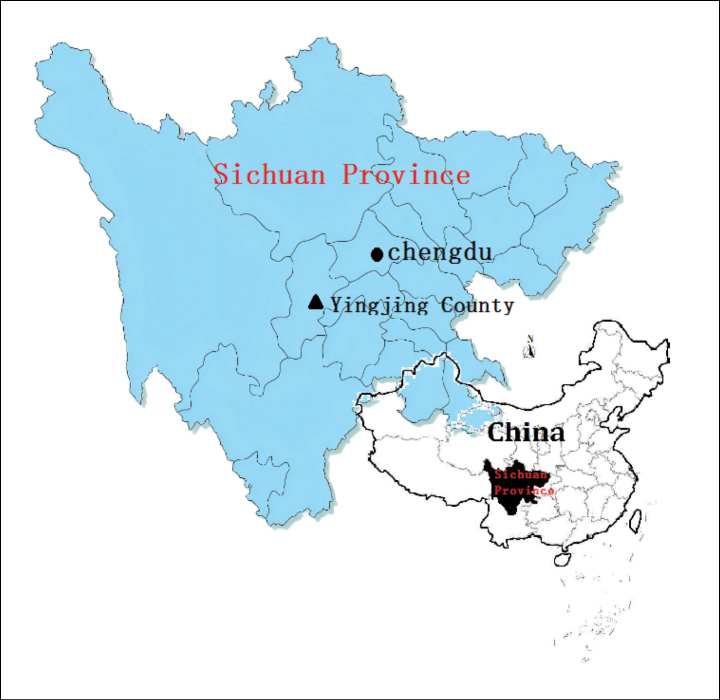
Map of Sichuan and China, the black triangle showing the location of *I. longcanggouensis*.

*Impatiens longcanggouensis* is restricted to sparse forests at elevations of 1100–2050 m in Longcanggou Town, Niubeishan Town, Siping Township and Xinjian Township, Yingjing County, Sichuan Province, China (Fig. 4).

#### Conservation status.

*Impatiens longcanggouensis* is preliminarily assessed as Near Threatened (NT) following the IUCN Red List Categories and Criteria (version 3.1). It is endemic to Yingjing County, Sichuan, with an estimated Extent of Occurrence (EOO) of approximately 1,000 km^2^. The species occurs in ecologically intact forest understorey across four townships at 1,100–2,050 m a.s.l., where over 20 subpopulations and more than 500 mature individuals have been documented. Although its relatively small range and population size approach the thresholds for Vulnerable under Criteria B (small distribution range combined with fragmentation, decline or extreme fluctuations) and D (very small population or very restricted distribution), no severe ongoing decline or imminent threats were observed. However, its restricted distribution and limited number of locations make it potentially susceptible to future threats, warranting monitoring under the Near Threatened category.

## Discussion

Morphologically, in addition to its close affinity with *I. recurvicornis*, the new species also resembles several Chinese endemic species including *I. trigonosepala*, *I. minimisepala* and *I. membranifolia*. These species share the following features: annual erect herbs with elongated inflorescences bearing one to three flowers; pedicels with basal bracts; small to medium-sized flowers; two lateral sepals; basal lobes of the lateral united petals not acuminate or extended into long filamentous hairs; acute anthers; and capsules that are elongated, fusiform, clavate or linear-cylindrical. The new species can be distinguished from these relatives by the diagnostic characteristics summarised in Table 1.

**Table 1.** Morphological comparison of longcanggouensis sp. nov. and four similar species.

Character	*I. longcanggouensis*	*I. recurvicornis*	*I. trigonosepala*	*I. minimisepala*	*I. membranifolia*
Leaf shape and size	lanceolate or ovate-lanceolate, 4–16 × 1–3.5(–4) cm	ovate-lanceolate or lanceolate, 5–8 × 2–2.5 cm	oblong or elliptic, 5–10 × 2–3 cm	ovate-lanceolate, 3–5 × 2–2.5 cm	ovate or ovate-oblong, 4–8 × 2.5–3.5 cm
Leaf base	without glands	often with two glands	with two stalked glands	with two glands	with two stalked glands
Leaf margin	shallowly arcuate-serrate	crenate-serrulate	coarsely crenate	serrulate	serrulate or crenate
Adaxial surface	hispid	glabrous	glabrous	glabrous	glabrous
Abaxial leaf colour	purplish-red, rarely pale green	pale green	pale green	pale green	pale green
Pedicel	4–10 cm long, filamentous	5–7 cm long, filamentous	3–5 cm long, filamentous	1–1.5 cm long, filamentous	ca. 2 cm long, filamentous
Inflorescence	two-flowered	one-flowered	one-flowered	two-flowered	two to five-flowered
Flower colour	white or light pink	pinkish-purple	pink	yellow	white
Flower size	1.2–1.8 cm long	3–4 cm long	ca. 2.5 cm long	ca. 1 cm long	ca. 1 cm long
Bract	lanceolate, 2–2.5 mm long, beaked apex, persistent	ovate, 3–4 mm long, apex aristate-acuminate tip, deciduous	lanceolate	very small, persistent	linear-lanceolate, 3–4 mm, mid-vein green
Lateral sepals	ovate or triangular-ovate, 2.5–3.5 mm long, mid-vein sometimes marginal	semi-ovate, ca. 7 mm long	triangular, 6 × 3 mm, mid-vein marginal	ovate, < 1 mm long	ovate, 3 mm long
Dorsal petal	orbicular, 6–8 mm diameter, abaxial mid-rib thickened with two erect, stout rostella	orbicular, 13 mm diameter, abaxial mid-rib thickened, apex beak-like	orbicular or oblate, 15 mm diameter, abaxial mid-rib simple or with a narrow crest	oblate, 10 mm wide, abaxial mid-rib with central acute ridge	orbicular, 4–5 mm diameter, abaxial mid-rib thickened with circular crest and hook-like apex
Lateral united petals	Sub-clawed, 10–15 mm long; basal lobes broadly ovate; distal lobes rhombic or oblong-dolabriform	Sub-clawed, 18–20 mm long; basal lobes oblong-flattened; distal lobes oblong	Sub-clawed, 14–16 mm long; basal lobes small; distal lobes obovate or obcordate	not clawed, 13–15 mm long; basal lobes orbicular; distal lobes obovate-orbicular or semilunar	not clawed, 8 mm long, narrow; basal lobes orbicular; distal lobes narrowly dolabriform
Lower sepal	narrowly funnel-form, 1.2–1.5 cm long; mouth spreading; base gradually narrowed into an incurved long spur	navicular, 1.3–1.5 cm deep; mouth oblique; base gradually narrowed into an incurved spur	navicular or funnel-form; mouth vertical; base gradually narrowed into an incurved spur 2–3 cm long	Funnel-form or cupular; ≤ 1 cm long; mouth vertical; base gradually narrowed into an incurved spur 2.5 cm long	navicular; mouth oblique; base abruptly narrowed into a slender 3 cm spur
Anthers	acute	acute	acute	acute	acute
Capsule	clavate, 1.5–2.3 cm long	narrowly linear, 2.5 cm long	linear, ca. 2 cm long	no record	linear-lanceolate, 9 mm long

*Impatiens longcanggouensis* is morphologically similar to *I trigonosepala*. However, it can be clearly distinguished by the following combination of characters: leaf shape lanceolate or ovate-lanceolate (vs. oblong or elliptic); leaf margin shallowly arcuate-serrate (vs. coarsely crenate); adaxial leaf surface hispid (vs. glabrous); abaxial leaf surface often purplish-red, rarely pale green (vs. pale green); inflorescence commonly two-flowered (vs. one-flowered); flowers 1.2–1.8 cm long (vs. ca. 2.5 cm long); and lower sepal narrowly funnel-form (vs. navicular or funnel-form).

*Impatiens longcanggouensis* is also morphologically similar to *I. minimisepala*, yet it can be clearly distinguished by the following combination of characters: stems often branched (vs. simple), leaves 4–16 cm long with shallowly arcuate-serrate margins and often purplish-red abaxial surfaces (vs. 3–5 cm long with serrulate margins and pale green abaxial surfaces), peduncles 4–40 cm long (vs. 1–1.5 cm long) and flowers 1.2–1.8 cm long, white to pale pink (vs. ca. 1 cm long, yellow).

*Impatiens longcanggouensis* is also morphologically similar to *I. membranifolia*, yet it can be distinguished by the following combination of characters: leaf shape lanceolate or ovate-lanceolate (vs. ovate or ovate-oblong); leaf margin shallowly arcuate-serrate (vs. serrulate or crenate); adaxial leaf surface hispid (vs. glabrous); abaxial leaf colour often purplish-red, rarely pale green (vs. pale green); peduncle 4–10 cm long (vs. ca. 2 cm long); inflorescence two-flowered (vs. two- to five-flowered); flower size 1.2–1.8 cm long (vs. ca. 1 cm long); dorsal petal 6–8 mm in diameter (vs. orbicular, 4–5 mm in diameter); lateral united petals sub-clawed, 10–15 mm long with distal lobes rhombic or oblong-dolabriform (vs. not clawed, 8 mm long with distal lobes narrowly dolabriform); and lower sepal narrowly funnel-form with a spreading mouth (vs. navicular with an oblique mouth).

*Impatiens longcanggouensis* is currently known only from Yingjing County, Sichuan Province. In contrast, its four morphologically similar relatives are distributed in distinct regions: *I. recurvicornis* and *I. membranifolia* occur in Chongqing Municipality and Hubei Province, *I. trigonosepala* in Chongqing Municipality and *I. minimisepala* in Yunnan Province. This clear geographical isolation precludes any potential gene flow. Together with a suite of stable morphological distinctions — namely, lanceolate or ovate-lanceolate leaves with shallowly arcuate-serrate margins and often purplish-red abaxial surfaces, a dorsal petal bearing two erect, stout rostella abaxially, the distal lobe of the lateral united petals with a small apical mucro and a narrowly funnel-form lower sepal — this isolation provides strong support for recognising it as a distinct biological species.

Based on its oblate-spheroidal or subprolate pollen grains with four germinal furrows, clavate capsules, five carpels and two-flowered inflorescences, *Impatiens longcanggouensis* is assigned to *Impatiens* subgen. *Impatiens* sect. (Yu et al. 2016).

## Supplementary Material

XML Treatment for Impatiens longcanggouensis

## Additional information

### Use of AI

No use of AI was reported.

### Author ORCIDs

Qiang Luo https://orcid.org/0000-0001-5043-2539

Hong Chen https://orcid.org/0000-0002-7745-9069

Ying Yuan https://orcid.org/0000-0002-2237-3452

Hong Yang https://orcid.org/0000-0002-9060-7708

Chan Liu https://orcid.org/0009-0005-1429-5692

Xinqiang Song https://orcid.org/0009-0008-9818-2493

Shen Ma https://orcid.org/0009-0007-4350-7345

### Data availability

All of the data that support the findings of this study are available in the main text.
